# Association Between Social Media Use and Patients’ Choice of Medical Practitioners Among the General Population in Saudi Arabia: A Cross-Sectional Study

**DOI:** 10.3390/healthcare13222870

**Published:** 2025-11-11

**Authors:** Nahla H. Hariri, Asayel T. Alruwais, Wafa M. Sodagar, Nada M. Allhaiby, Tasneem M. Moglan, Lina I. Kinkar, Raneem F. Alskhairi, Fatima A. Almekhlafi, Asia M. Kalantan, Ruba F. Mohammed, Rawan Aljuwaybiri, Nizar S. Bawahab, Saleh A. K. Saleh, Heba M. Adly

**Affiliations:** 1 Department of Community Medicine and Pilgrims Healthcare, College of Medicine, Umm Al-Qura University, Makkah 21955, Saudi Arabia; 2 Maternity and Children Hospital Makkah, Makkah 24246, Saudi Arabia; atalruwais@gmail.com; 3 King Faisal Hospital, Makkah Health Cluster, Ministry of Health, Makkah 24236, Saudi Arabia; wmsodagar@gmail.com; 4 Obstetrics and Gynecology Department, Makkah Health Cluster, Makkah 24342, Saudi Arabia; allhaibynda37@gmail.com; 5 Independent Researcher, College of Medicine Graduate, Umm Al-Qura University, Makkah 21955, Saudi Arabiaasia.klntn38@gmail.com (A.M.K.); 6 Family Medicine Academy, Makkah Health Cluster, Makkah 24342, Saudi Arabia; lleenaik@gmail.com (L.I.K.); fatima79x@gmail.com (F.A.A.); 7 Department of Internal Medicine, King Faisal Hospital, Makkah 24236, Saudi Arabia; r2neemat@gmail.com; 8 Department of General Surgery, Hera General Hospital, Makkah 24227, Saudi Arabia; rubaf53@gmail.com; 9 Alnawarryah Primary Health Care Centre, Makkah 24236, Saudi Arabia; rawan.aljuwaybiri@gmail.com; 10 Department of General Surgery, King Faisal Hospital, Makkah 24236, Saudi Arabia; nbawahab@moh.gov.sa; 11 Directorate of Institutional Excellence, Batterjee Medical College, Jeddah 21442, Saudi Arabia; saleh.abdrabuh@bmc.edu.sa

**Keywords:** social media, patients’ choices, medical practitioner, Saudi Arabia

## Abstract

**Background:** Social media is increasingly shaping patient decision-making about the choice of healthcare providers. However, its role in the Saudi context remains underexplored. This study aimed to examine the association between social media use and patient decision-making regarding the choice of healthcare providers in Saudi Arabia. **Methods:** This cross-sectional study used a validated online questionnaire. The study was conducted between December 2023 and May 2024 to assess demographics, social media usage, and decision-making factors. **Results:** 1242 participants completed the survey; most participants (96.2%) had personal social media accounts. Instagram (41.3%) and X (37.6%) were the preferred platforms to look up or follow doctors. The most influential factors in choosing a healthcare provider included physician qualifications, online reviews, and patient testimonials. While 81.3% believed medical practices should maintain a social media presence, traditional factors remained more decisive than promotional content. Younger participants, females, and those in the health field were significantly more likely to follow doctors online (*p* < 0.001). **Conclusions:** Social media plays a notable role in patient decision-making about healthcare provider selection in Saudi Arabia, particularly among younger individuals and those working in the health sector. Nevertheless, clinical qualifications and trust indicators remain paramount. These findings support the need for healthcare professionals to maintain a credible, ethical, and informative digital presence to enhance patient engagement and informed decision-making.

## 1. Introduction

Social media platforms are internet-based tools that enable users to create, share, and engage with content across blogs, professional and social networks, photo/video sharing, review sites, bookmarking, social gaming, and virtual worlds. Platforms such as Facebook, X, Instagram, YouTube, Flickr, and LinkedIn have expanded rapidly [1]. An increasing proportion of the public now turns to these tools for health-related decisions [2]. These channels facilitate rapid exchange of medical information that patients can access directly [3], provide informal mechanisms for illness reporting and peer connection, and expose users to external opinions that shape clinical decision-making; teleconsultation further reduces geographic barriers and may lower costs for some users [4,5].

In healthcare, social media can strengthen peer support, enhance public-health monitoring, and improve access to reliable information [6]. At the same time, concerns persist regarding governance, content quality and accuracy, patient privacy and confidentiality, blurred personal–professional boundaries, liability, and methodological rigor in social-media–based research [7]. Collectively, these considerations indicate that social media factors may function as decision cues—including platform credibility, online reviews/ratings, and visible credentials—that can be evaluated in relation to patients’ provider selection [6,7].

Within the Arab region, and in Saudi Arabia specifically, there is documented use of social platforms for professional development, public-facing health communication, and service navigation [1,8]. Building on this context, the present study focuses on predefined decision dimensions that are examined later in the Materials and Methods/Results: (i) preferred platforms; (ii) credibility signals (online reviews/ratings, visible qualifications); (iii) promotional content versus authenticity; (iv) access/logistics features (appointment convenience, e-prescriptions); and (v) subgroup differences (age, sex, region, health insurance, health-sector affiliation) [1,2,3,8].

Outside Saudi Arabia, studies from North America and Europe report that online ratings/reviews, visible credentials, and responsiveness on platforms such as Facebook, X, Instagram, and YouTube are associated with patients’ selection and switching of providers [9,10]. Evidence from the broader MENA region likewise shows a high reliance on peer recommendations and platform credibility, particularly among younger users [11]. Recent reviews further note that content quality and source transparency shape trust and choice, whereas promotional bias and misinformation may distort decisions; privacy concerns and confidence in official channels can moderate these effects [12,13]. Accordingly, this study aimed to examine the association between social media and patient decision-making about healthcare provider choice in Saudi Arabia, and to identify which platforms, content types, and practitioner attributes are most strongly associated with booking decisions.

## 2. Materials and Methods

### 2.1. Design and Setting of the Study

The study was conducted between December 2023 and May 2024 using a web-based online survey that was distributed nationwide across all regions of Saudi Arabia.

This was an observational cross-sectional survey; analyses were descriptive and bivariate, examining associations rather than causal effects. Any use of the term “influence” is descriptive and does not imply causality. As this was an online non-probability convenience sample of volunteers recruited via social media; the sample is not statistically representative of the Saudi population.

### 2.2. Participant Characteristics and Sampling Strategy

The study aimed to gather insights from individuals aged 18 years and older, including both Saudi citizens and non-citizen residents. To ensure broad participation and accessibility, the survey link was distributed and regularly reposted on popular social media platforms, including Facebook, X, Snapchat, and Instagram. We did not apply quotas or post-stratification weights; therefore, inferences generalize primarily to Arabic/English-speaking online adults who chose to participate.

A convenience sampling strategy was employed. Participation in the survey was entirely voluntary. At the start of the online questionnaire, a detailed overview of the study’s objectives and procedures was provided. Participants were informed that the survey was anonymous, and that no personally identifiable information would be collected. It was clearly stated that all responses would be used strictly for academic research purposes. By proceeding with the survey, individuals implicitly provided informed consent. The data were handled in accordance with ethical research standards and securely stored to maintain confidentiality.

### 2.3. Questionnaire Tool

To fulfill the study objectives, a structured questionnaire previously developed and validated by Fatollahi et al., 2020 [14] was employed. The instrument consisted of five main sections. The first section contained a consent statement to secure voluntary participation. The second section recorded the data collector’s identification code to enable tracking and quality assurance. The surveyor code identified distribution channel only (e.g., link source) for quality monitoring. It was not linked to personal identity, and analyses used fully anonymized data. The third section captured socio-demographic variables, including age, gender, and educational attainment. The fourth section explored participants’ engagement with social media, including whether they had active accounts, the most frequently used platforms, browsing habits related to healthcare providers’ websites, and preferences for using social media to search for medical practitioners. Additionally, this section assessed participants’ attitudes regarding the necessity for medical practices to maintain an online social media presence. Primary outcome (Social-media influence on provider choice) was defined as [binary: Yes/No] based on the item, ‘Did information encountered on social media influence your choice of healthcare provider for your most recent booking?’ (Yes/No). The fifth and final section examined various factors that may influence individuals’ selection of healthcare providers. These included the presence of clinics on social media, website usability, quality of online reviews, accessibility to national health services, and availability of advanced medical technologies. Participants were also asked to evaluate other influential factors such as personal recommendations, physician credentials, review positivity, awards, originality of online content, before-and-after images, promotional offers, and the volume of social media engagement (e.g., likes). Furthermore, the questionnaire addressed perceptions regarding the effectiveness of social media marketing in attracting new patients, its return on investment relative to traditional advertising methods, and preferences for booking procedures such as cosmetic or otherwise with social media-prominent physicians. The questionnaire was administered in Arabic and English. We performed forward-translation (English to Arabic) and back-translation by independent bilingual translators. A pilot (n = 30) tested administration flow and timing. For multi-item constructs, internal consistency was evaluated using Cronbach’s α.

### 2.4. Data Collection Process

The data collection process was conducted through the dissemination of a structured, pre-validated questionnaire using multiple social media platforms, including Facebook, Instagram, WhatsApp, and X The survey was administered via Google Forms, and participants were encouraged to share the link within their networks to maximize reach and enhance response rates. To maintain visibility, the survey invitation was reposted at regular weekly intervals throughout the data collection period.

Each participant received a cover letter detailing the study’s aims, emphasizing voluntary participation, and ensuring confidentiality and data protection. No financial or material incentives were offered. Respondents retained the right to discontinue participation at any stage during the completion of the questionnaire without consequence.

At the beginning of the survey, participants were presented with a binary (yes/no) consent statement: “Do you agree to have your responses used for the purpose of writing a scientific article?” Only those who responded affirmatively were included in the final analysis. Responses submitted without consent were excluded. The survey process was anonymous, with no identifiable personal information collected, and conformed to Google’s privacy and data security policies.

To protect data quality, the form used one-submission per device (cookies), reCAPTCHA, and an attention-check item (‘Please select “Agree” for this item’). We recorded server timestamps to screen for implausible completion times.

### 2.5. Sample Size Calculation

This survey used a precision-based calculation for a single proportion as the primary parameter. We assumed a conservative expected proportion *p* = 0.50 (maximizes required n), two-sided α = 0.05 (Z = 1.96), and absolute precision d = 0.05. Using n = Z^2^·p(1 − p)/d^2^, the minimum sample was n = 384; we set n = 400 to allow for non-response, consistent with standard guidance for cross-sectional surveys [15]. As a secondary justification for subgroup comparisons, the achieved sample (n = 1514) provides >90% power (α = 0.05, two-sided) to detect small-to-moderate differences in proportions in chi-square tests (approximately Cohen’s w = 0.12–0.15). Subgroup comparisons were planned only where cell counts ≥10 per category; categories with sparse cells were collapsed a priori to protect estimate stability.

### 2.6. Ethical Approval

Ethical approval for the study was obtained from the Umm Al-Qura University (UQU) Research Ethics Committee in Makkah, Saudi Arabia (Approval No. HAPO-02-K-012-2023-02-1471). All procedures involving human participants were conducted in accordance with the ethical principles outlined in the “Declaration of Helsinki, as revised in 2013”.

### 2.7. Data Analysis

Data cleaning excluded: (i) duplicates (identical age/sex/region and near-identical timestamps), (ii) failed attention-check, (iii) completion time below 10 min (pre-specified minimum), and (iv) straight-lining across multi-item blocks. Sensitivity analyses repeated estimates with/without these exclusions. Analyses were descriptive and bivariate; no causal modeling was performed. The primary analysis modeled Pr (‘influenced’ = Yes). The data were collected, reviewed, and analyzed using the Statistical Package for Social Sciences (SPSS) version 21 (SPSS Inc., Chicago, IL, USA). Descriptive analysis was performed to present frequency distributions and percentages for variables such as participants’ socio-demographic data, social media use, and factors associated with doctor selection. Graphs were used to illustrate key findings, such as the most important factors for choosing a medical practice or evaluating its social media presence.

Cross-tabulation was applied to explore relationships between participants’ browsing habits, preferences for social media-famous doctors, and following behaviors on social media. Pearson Chi-square tests and exact probability tests were used to determine statistical significance, with a *p*-value of ≤0.05 considered significant.

Beyond descriptive and bivariate analyses, we fit multivariable logistic regression with the binary outcome (‘influenced’), reporting adjusted odds ratios (aOR) and 95% CIs. Covariates were age group, sex, region, education, insurance status, health-sector affiliation, preferred platform(s), and key credibility signals (reviews, credentials). Sensitivity analyses included: (i) restricting to respondents with completion time ≥10 min, (ii) excluding straight-liners, and (iii) alternative codings of platform variables.

Diagnostics included variance inflation factors (VIF < 5) for multicollinearity, Hosmer–Lemeshow goodness-of-fit, ROC AUC for discrimination, and cluster-robust standard errors by region.

## 3. Results

Of 1514 submissions, 272 were excluded per pre-specified criteria (duplicates, failed attention-check, completion time <10 min, straight-lining), leaving 1242 records for analysis. Among them, 415 (33.4%) were from the Eastern region, 356 (28.7%) from the Northern region, and the remainder from various other regions. Participants’ ages ranged from 18 to over 55 years, with a mean age of 27.9 ± 11.4 years. The majority of respondents were female 804 (64.7%), and most held a university degree 884 (71.2%). Additionally, 159 (12.8%) participants had a high school education, while 92 (7.4%) held a postgraduate degree.

Regarding health insurance, 685 (55.2%) participants used governmental services, 354 (28.5%) had private insurance, and 120 (9.7%) had no insurance coverage. Furthermore, 691 (55.6%) participants were either working or studying in the health field (Table 1).

As shown in Table 2, most participants 1195 (96.2%) reported having a personal social media account. The most used platforms were Instagram (32.8%), followed by X (23.9%), Snapchat (21.3%), and TikTok (17.4%). Overall, 68.7% (n = 853) reported browsing a doctor’s or clinic’s social media page/website. Within this group, 41.5% (n = 515) reported searching and following doctors, 20.7% (n = 257) searched only, and 37.8% (n = 470) never followed.

When asked about their preferred social media platform for finding doctors’ accounts, Instagram was the most popular choice (41.3%), followed by X (37.6%) and Snapchat (11.6%). Furthermore, 1010 participants (81.3%) agreed that medical practices should maintain an active presence on social media.

**Figure 1:**
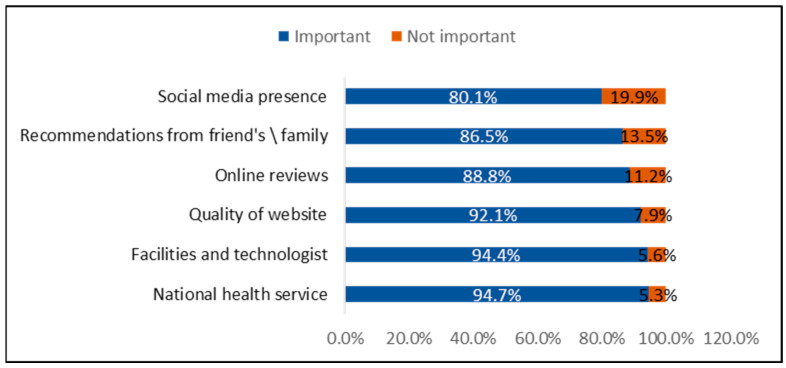
Key Factors Influencing Patients’ Selection of a Medical Practice (n = 1242).

As illustrated in Figure 1, the most important factors reported by patients when choosing a medical practice were the availability of national health services (94.7%), followed closely by the quality of facilities and technology (94.4%). Other significant factors included the quality of the practice’s website (92.1%), online reviews (88.8%), recommendations from friends or family (86.5%), and the practice’s social media presence (80.1%).

**Figure 2:**
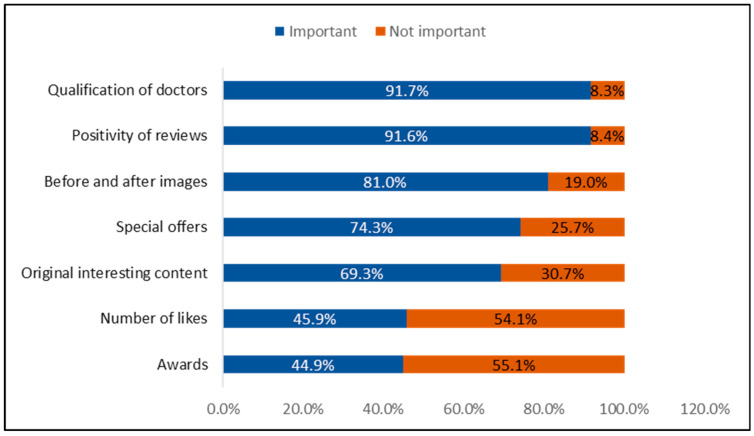
Key Factors Influencing Patients’ Evaluation of a Medical Practice’s Social Media Presence. (n = 1242).

Figure 2 illustrates the factors most important to patients when reviewing a medical practice’s social media account. The top factors include the qualification of doctors (91.7%), positivity of reviews (91.6%), before-and-after images (81%), special offers (74.3%), original and engaging content (69.3%), the number of likes (45.9%), and awards (44.9%).

Table 3 presents the participants’ views on the role of social media in selecting a doctor. A total of 1153 participants (92.9%) believe that social media is effective for attracting new patients to a medical practice, while 1070 participants (86.2%) think the return on investment from social media marketing is higher than that from traditional marketing. Regarding content preferences, 525 participants (42.3%) prefer purely medical content on doctors’ social media accounts, 345 participants (27.8%) favor accounts that include the doctor’s lifestyle, and 270 participants (21.8%) reported no preference. Additionally, 492 participants (39.6%) prefer doctors who are famous on social media over those who are not, 411 participants (33.1%) consider a doctor’s social media fame when booking a cosmetic procedure, and 393 participants (31.7%) base their decision on social media fame when booking for non-cosmetic health concerns.

Table 4 presents the factors considered by participants before and during booking an appointment with a doctor. Because respondents could select more than one factor, percentages exceed 100%. Before booking, the most important factors were patient feedback (34.6%), CV (Curriclum Vitae) (18.9%), before-and-after images (12.8%), explanatory clarity (9.8%), clinic tools (6.4%), fame/followers (2.6%), do not search (14.7%). During booking the most considered factors included nearest appointment (68.7%), reviews (57.0%), advice of family/friends (52.8%), CV (40.9%), popularity (23.8%), advertisement (11.8%).

**Figure 3:**
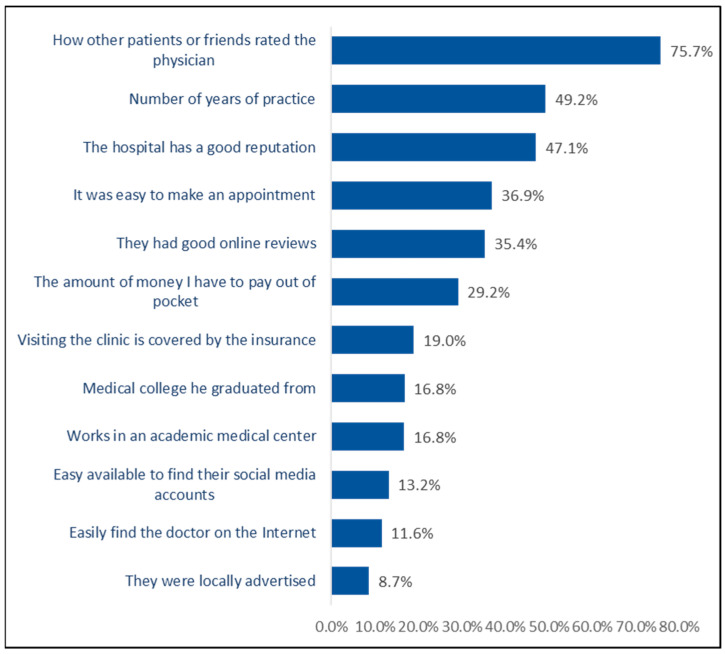
Factors prioritized when selecting a treating physician (overall importance) (n = 1242).

Figure 3 shows the factors most highly rated by participants when selecting a treating physician. The most significant factors included: “How other patients or friends rated the physician” (75.7%), “Number of years of practice” (49.2%), “The hospital has a good reputation” (47.1%), “It was easy to make an appointment” (36.9%), “They had good online reviews” (35.4%), and “The amount of money I have to pay out of pocket” (29.2%). The least rated factors were “Easily find the doctor on the Internet” (11.6%) and “They were locally advertised” (8.7%).

Table 5 presents the factors associated with participants’ browsing of social media or websites for doctors or clinics. Browsing rates were highest in the Central (83.8%) and Northern (75.0%) regions, higher than the Western (67.4%) and Southern (61.3%) regions (*p* < 0.001). Additionally, 71.9% of female participants browsed social media for doctors, compared to 62.8% of male participants (*p* = 0.001). Furthermore, 76.6% of participants working or studying in the health field browsed social media, compared to 58.8% of those in other fields (*p* = 0.001). Browsing for doctor selection was also more common among those with a personal social media account (69.9%) compared to those without (38.3%) (*p* = 0.001).

Table 6 shows the factors associated with participants’ preference for famous social media doctors. Preference was highest in the Central and Southern regions compared to those from other regions (*p* = 0.001). Additionally, Preference for famous social-media doctors did not increase with education; respondents with a higher degree were more likely to report ‘No’ (65.2%) than ‘Yes’ (34.8%) (*p* = 0.049).

Table 7 shows the factors associated with participants’ following of doctors on social media. Following doctors was significantly higher among younger participants who searched for and followed doctors, compared to older participants (*p* = 0.001). Additionally, 47.5% of female participants searched for and followed doctors on social media, compared to 30.4% of males (*p* = 0.001). Following doctors was reported by 47.6% of those working or studying in the health field, compared to 33.8% of others (*p* = 0.001). Furthermore, 42.9% of those with private insurance searched for and followed doctors on social media, compared to 33.7% of those without private insurance (*p* = 0.033). Lastly, 42.5% of participants with a personal social media account searched for and followed doctors, compared to 14.9% of those without an account (*p* = 0.001).

## 4. Discussion

This cross-sectional survey of 1242 adults in Saudi Arabia examined how social media relates to patients’ choice of healthcare providers, focusing on platform use, content cues, and practitioner attributes that shape decisions. Consistent with global trends, visually oriented networks (e.g., Instagram, Snapchat, TikTok) were the most salient environments for pre-visit engagement, while X, YouTube, and LinkedIn remained relevant in specific use cases. These patterns mirror international observations that social media has become a routine channel for health communication and patient–clinician interaction, offering access, peer connection, and low-cost outreach, yet raising concerns about accuracy, privacy, and professional boundaries [2,5,16]. These patterns speak directly to our aim of identifying platform and credibility cues associated with provider selection in the Saudi context. The Saudi context is comparable, with documented integration of social media into professional development and public-facing health communication [1,17].

Participants prioritized credibility signals when evaluating clinicians online—particularly visible qualifications and independent reviews with outcome imagery and awards considered secondary. This hierarchy aligns with prior literature showing that verified expertise and third-party feedback serve as risk-reducing cues at the point of provider choice [3,5,18], while overt promotional content is generally less persuasive in clinical contexts [4,19,20,21,22]. At the same time, the prominence of before-and-after images warrants caution: reviews repeatedly note that de-contextualized visuals can introduce promotional bias and unrealistic expectations, underscoring the need for source transparency and appropriate clinical framing [19,20,21,22,23,24,25,26]. Together, these findings support guidance that foregrounds digital professionalism accurate information, clear scope of practice, and transparent provenance cover advertising-led strategies [6,7,19,20,25,26,27,28].

Beyond profile signals, participants valued service logistics such as convenient booking, e-prescriptions, and perceived service quality, which is consistent with the diffusion and uptake of national e-health solutions (Seha, Mawid, Tetamman) during and after COVID-19 [27,28,29,30,31]. This suggests that social media often functions as an entry point directing patients toward established, trusted digital pathways rather than substituting for them, an interpretation in line with reports of strong engagement with official channels during periods of heightened information need [31,32,33]. Peer inputs also remained influential: reliance on online reviews and family/friend recommendations echoes international evidence that digital reputation must be reinforced by real-world experience to sustain trust [19,20,21].

Subgroup patterns were coherent with prior work. Younger adults and women were more likely to browse or follow clinicians, and a preference for well-known “social-media doctors” appeared more often in certain regions and among higher-education groups, paralleling documented demographic and contextual gradients in digital health uptake and trust formation [19,34,35,36]. Content preferences provide a practical lever: the tendency to favor medical-only content over mixed personal material is consistent with evidence that accuracy, source transparency, and measured tone underpin online credibility [6,22,23,37,38,39,40,41]. Overall, the results support a segmented communication approach concise, visual formats coupled with explicit credentialing and clear links to authoritative services tailored to audience characteristics.

Contemporary guidance on digital professionalism emphasizes separating personal and professional identities, transparent sourcing, and accurate, clinically framed content principles that align with participants’ preference for credentials and medical-only posts [42]. Concurrent evidence links perceived health misinformation on social media to lower trust in healthcare and highlights practical moderation approaches to curb engagement with misleading posts [43,44]. Within Saudi Arabia and comparable regional contexts, specialty-focused studies show that professional knowledge posts and authentic engagement (rather than overt promotion) positively shape patient perceptions and adherence [45]. At the system level, analyses of public-health communication demonstrate sustained engagement with official channels during periods of heightened information need, supporting our recommendation to link profiles to authoritative services rather than rely on marketing alone [46]. Our findings should be interpreted as associative signals in a non-probability online sample; confirmatory, longitudinal or experimental designs are needed to test causal mechanisms

Study Limitations: Because the survey was disseminated via social media, the sample disproportionately comprised active social-media users, introducing selection bias and likely inflating exposure to online cues relative to the general population The cross-sectional, self-reported design permits inference about associations rather than causation and reflects perceived influence rather than verified booking behavior. Although a previously used instrument framework was employed, psychometric properties were not re-established in this dataset, and linkage to transactional data (e.g., appointment logs, Ministry of Health application analytics) was not available. Regional participation and occupational mix varied, which may affect representativeness. These constraints should be considered when interpreting the findings and planning confirmatory research.

### Future Directions

This study confirms the significant role social media plays in shaping healthcare decisions in Saudi Arabia, particularly among younger, educated, and digitally engaged demographics. However, while patients value online presence, qualifications, peer reviews, and professionalism remain decisive factors. Future research should investigate why some patients prefer non-famous doctors despite their limited online activity and examine barriers such as affordability, convenience, and perceived cultural compatibility in more detail.

## 5. Conclusions

This study assessed how social media influences the selection of medical practitioners among the general population in Saudi Arabia. The results demonstrated that a significant proportion of participants actively use platforms such as Instagram and X to search for and follow healthcare providers, with physician qualifications, online reviews, and visual content (before-and-after images) being the most influential criteria. Younger individuals, females, and those working or studying in the health sector were more likely to rely on social media when choosing a practitioner. These findings highlight the growing importance of a professional digital presence in healthcare within Saudi Arabia. Future research in the Saudi context should further examine how digital health behaviors vary across regions and socioeconomic groups and evaluate these associations using designs capable of testing causality.

## Figures and Tables

**Table 1:** Socio-Demographic Characteristics of Study Participants in Saudi Arabia (n = 1242).

Socio-Demographics	n (%)
**Region**	
Central Region	167 (13.4%)
Northern region	356 (28.7%)
Eastern Region	415 (33.4%)
Western Region	92 (7.4%)
Southern Region	212 (17.1%)
**Age in years**	
18–25	468 (37.7%)
26–35	460 (37.0%)
36–45	165 (13.3%)
46–55	113 (9.1%)
>55	36 (2.9%)
**Gender**	
Male	438 (35.3%)
Female	804 (64.7%)
**Educational level**	
Below high school	16 (1.3%)
High school	159 (12.8%)
Diploma	91 (7.3%)
College degree	884 (71.2%)
Higher degree	92 (7.4%)
**Health insurance**	
No insurance	120 (9.7%)
Student	83 (6.7%)
Government services	685 (55.2%)
Private	354 (28.5%)
**Do you work or study in the health field?**	
Yes	691 (55.6%)
No	551 (44.4%)

**Table 2:** Patterns and Types of Social Media Use and Physicians Followed by Study Participants in Saudi Arabia (n = 1242).

Social Media Use	n (%)
**Do you have a personal social media account?**	
Yes	1195 (96.2%)
No	47 (3.8%)
**What is the most used social media platform you use?**	
Instagram	407 (32.8%)
X	297 (23.9%)
Snapchat	264 (21.3%)
Tiktok	216 (17.4%)
Facebook	33 (2.7%)
LinkedIn	25 (2.0%)
**Have you ever browsed (Facebook page, X, Instagram, Snapchat, LinkedIn) or the website of your doctor or clinic?**	
Yes	853 (68.7%)
No	389 (31.3%)
**Have you ever followed any of your doctors on social media?**	
Yes, searched and followed	515 (41.5%)
Yes, but only searched	257 (20.7%)
Never	470 (37.8%)
**What is your preferred social networking site where you are looking for your doctor’s account?**	
Instagram	513 (41.3%)
X	467 (37.6%)
Snapchat	144 (11.6%)
Facebook	71 (5.7%)
LinkedIn	47 (3.8%)
**Medical practice should have a social media presence.**	
Agree	1010 (81.3%)
Disagree	232 (18.7%)

**Table 3:** Participants’ Perception of social media and Its Role in Choosing a Doctor (n = 1241).

Attitude	n (%)
**How effective is social media for a medical practice to attract new patients?**	
Effective	1153 (92.9%)
Ineffective	88 (7.1%)
**What do you think is the return on investment of social media marketing when compared to conventional marketing for a medical practice?**	
Higher	1070 (86.2%)
Lower	171 (13.8%)
**Do you prefer doctors’ social media accounts that include his/her lifestyle?**	
Yes, I do	345 (27.8%)
No difference	270 (21.8%)
No, I prefer pure medical content	525 (42.3%)
I do not follow doctors	101 (8.1%)
**Do you prefer famous social media doctors over non-social media famous?**	
Yes	492 (39.6%)
No	749 (60.4%)
**I depend on how famous the doctor is on social media when booking a cosmetic procedure**	
Yes	411 (33.1%)
No	285 (23.0%)
I do not care about cosmetics	545 (43.9%)
**I depend on how famous the doctors are on social media when booking for a disease that is not cosmetic**	
Yes	393 (31.7%)
No	469 (37.8%)
I do not care	379 (30.5%)

Denominator is 1241 due to one missing response (item nonresponse).

**Table 4:** Factors Considered (multiple responses allowed) by Study Participants Before and During the Appointment Booking Process with a Doctor (n = 772).

Booking	n (%)
**If you search your doctors on social media before booking, what is the most important factor you look for?** (select all that apply)	
The feedback of his patients	430 (34.6%)
CV	235 (18.9%)
Before and after pictures of his patients	159 (12.8%)
The way he/she explains diseases	122 (9.8%)
The quality of the tools used in the clinic	80 (6.4%)
If he/she is famous and the number of followers	32 (2.6%)
I do not search my doctors on social media	183 (14.7%)
**What is the most important factor when booking an appointment with a doctor?** (select all that apply)	
Nearest appointment	852 (68.7%)
Reviews of previous patients	707 (57.0%)
The advice of family or Friend	655 (52.8%)
CV	508 (40.9%)
How popular is the doctor on social media	295 (23.8%)
Advertisement on social media	147 (11.8%)

Note: Multiple responses allowed; percentages sum to >100%. Denominators: pre-booking items among respondents who reported searching before booking (n = 772); during-booking items among all respondents (n = 1242).

**Table 5:** Factors Associated with Participants’ Browsing of Social Media or Websites for Doctors or Clinics.

Factors	Have You Ever Browsed Social Media or the Website of Your Doctor or Clinic?		*p*-Value
	Yes	No	
	n (%)	n (%)	
**Residential region**			0.001 *
Central	140 (83.8%)	27 (16.2%)	
Northern	267 (75.0%)	89 (25.0%)	
Eastern	254 (61.2%)	161 (38.8%)	
Western	62 (67.4%)	30 (32.6%)	
Southern	130 (61.3%)	82 (38.7%)	
**Age in years**			0.010 *
18–25	328 (70.1%)	140 (29.9%)	
26–35	333 (72.4%)	127 (27.6%)	
36–45	106 (64.2%)	59 (35.8%)	
46–55	65 (57.5%)	48 (42.5%)	
>55	21 (58.3%)	15 (41.7%)	
**Gender**			0.001 *
Male	275 (62.8%)	163 (37.2%)	
Female	578 (71.9%)	226 (28.1%)	
**Education level**			0.064
Below high school	10 (62.5%)	6 (37.5%)	
High school	98 (61.6%)	61 (38.4%)	
Diploma	55 (60.4%)	36 (39.6%)	
College degree	627 (70.9%)	257 (29.1%)	
Higher degree	63 (68.5%)	29 (31.5%)	
**Do you work or study in the health field?**			0.001 *
Yes	529 (76.6%)	162 (23.4%)	
No	324 (58.8%)	227 (41.2%)	
**Health insurance**			0.224
No insurance	77 (64.2%)	43 (35.8%)	
Student	54 (65.1%)	29 (34.9%)	
Governmental	465 (67.9%)	220 (32.1%)	
Private	257 (72.6%)	97 (27.4%)	
**Do you have a personal social media account?**			0.001 *^,^^
Yes	835 (69.9%)	360 (30.1%)	
No	18 (38.3%)	29 (61.7%)	

*p*: Pearson X2 test; ^: Exact probability test, * *p* < 0.05 (significant).

**Table 6:** Factors Associated with Participants’ Preference for Famous Social Media Doctors (n = 1241).

Factors	Do You Prefer Famous Social Media Doctors over Non-Social Media Famous Ones?		*p*-Value
	Yes	No	
	n (%)	n (%)	
**Residential region**			0.001 *^,^^
Central	87 (52.1%)	80 (47.9%)	
Northern	155 (43.5%)	201 (56.5%)	
Eastern	128 (30.8%)	287 (69.2%)	
Western	28 (30.4%)	64 (69.6%)	
Southern	94 (44.5%)	117 (55.5%)	
**Age in years**			0.122
18–25	185 (39.5%)	283 (60.5%)	
26–35	166 (36.1%)	294 (63.9%)	
36–45	73 (44.2%)	92 (55.8%)	
46–55	49 (43.8%)	63 (56.3%)	
>55	19 (52.8%)	17 (47.2%)	
**Gender**			0.738
Male	176 (40.3%)	261 (59.7%)	
Female	316 (39.3%)	488 (60.7%)	
**Education level**			0.049 *^,^^
Below high school	8 (50.0%)	8 (50.0%)	
High school	73 (45.9%)	86 (54.1%)	
Diploma	45 (49.5%)	46 (50.5%)	
College degree	334 (37.8%)	549 (62.2%)	
Higher degree	32 (34.8%)	60 (65.2%)	
**Do you work or study in the health field?**			0.245
Yes	264 (38.2%)	427 (61.8%)	
No	228 (41.5%)	322 (58.5%)	
**Health insurance**			0.070
No insurance	35 (29.2%)	85 (70.8%)	
Student	35 (42.2%)	48 (57.8%)	
Governmental	271 (39.6%)	413 (60.4%)	
Private	151 (42.7%)	203 (57.3%)	
**Do you have a personal social media account?**			0.678
Yes	472 (39.5%)	722 (60.5%)	
No	20 (42.6%)	27 (57.4%)	

*p*: Pearson X2 test; ^: Exact probability test, * *p* < 0.05 (significant). Denominator is 1241 due to one missing response (item nonresponse).

**Table 7:** Factors Associated with Participants’ Following of Doctors on Social Media (n = 1242).

Factors	Have You Ever Followed Any of Your Doctors on Social Media?			*p*-Value
	Yes, Searched and Followed	Yes, but Only Searched	Never	
	n (%)	n (%)	n (%)	
**Residential region**				0.096
Central	87 (52.1%)	41 (24.6%)	39 (23.4%)	
Northern	166 (46.6%)	69 (19.4%)	121 (34.0%)	
Eastern	163 (39.3%)	78 (18.8%)	174 (41.9%)	
Western	38 (41.3%)	24 (26.1%)	30 (32.6%)	
Southern	61 (28.8%)	45 (21.2%)	106 (50.0%)	
**Age in years**				0.001 *
18–25	197 (42.1%)	115 (24.6%)	156 (33.3%)	
26–35	199 (43.3%)	85 (18.5%)	176 (38.3%)	
36–45	74 (44.8%)	35 (21.2%)	56 (33.9%)	
46–55	37 (32.7%)	18 (15.9%)	58 (51.3%)	
>55	8 (22.2%)	4 (11.1%)	24 (66.7%)	
**Gender**				0.001 *
Male	133 (30.4%)	102 (23.3%)	203 (46.3%)	
Female	382 (47.5%)	155 (19.3%)	267 (33.2%)	
**Education level**				0.230 ^
Below high school	4 (25.0%)	1 (6.3%)	11 (68.8%)	
High school	62 (39.0%)	29 (18.2%)	68 (42.8%)	
Diploma	38 (41.8%)	16 (17.6%)	37 (40.7%)	
College degree	370 (41.9%)	193 (21.8%)	321 (36.3%)	
Higher degree	41 (44.6%)	18 (19.6%)	33 (35.9%)	
**Do you work or study in the health field** **?**				0.001 *
Yes	329 (47.6%)	146 (21.1%)	216 (31.3%)	
No	186 (33.8%)	111 (20.1%)	254 (46.1%)	
**Health insurance**				0.033 *
No insurance	48 (40.0%)	24 (20.0%)	48 (40.0%)	
Student	28 (33.7%)	25 (30.1%)	30 (36.1%)	
Governmental	287 (41.9%)	122 (17.8%)	276 (40.3%)	
Private	152 (42.9%)	86 (24.3%)	116 (32.8%)	
Do you have a personal social media account?				0.001 *^,^^
Yes	508 (42.5%)	251 (21.0%)	436 (36.5%)	
No	7 (14.9%)	6 (12.8%)	34 (72.3%)	

*p*: Pearson X2 test; ^: Exact probability test, * *p*-value < 0.05 (significant).

## Data Availability

The raw data supporting the conclusions of this article will be made available by the authors upon request. The dataset is not publicly available due to privacy and ethical restrictions, in accordance with institutional ethical approval.
